# Microbial network signatures of early colonizers in infants with eczema

**DOI:** 10.1002/imt2.90

**Published:** 2023-02-16

**Authors:** Liujing Huang, Guihua Pan, Yifei Feng, Zijing Fan, Kai Ma, Runxin Wang, Guangxian Wang, Guangye Huang, Sixia Huang, Yuhui Hou, Mulan Han, Liwei Xie, Ying Ma

**Affiliations:** ^1^ Obstetrics and Gynecology Medical Center, Zhujiang Hospital Southern Medical University Guangzhou China; ^2^ Guangdong Provincial Key Laboratory of Microbial Culture Collection and Application, State Key Laboratory of Applied Microbiology Southern China, Institute of Microbiology Guangdong Academy of Sciences Guangzhou China; ^3^ School of Public Health Xinxiang Medical University Xinxiang China; ^4^ Jiangsu New‐bio Biotechnology Co., Ltd. Jiangyin China

## Abstract

In this longitudinal cohort study, our results demonstrated that there are rhythmic changes in gut microbial network signatures in early life, and healthy infants adopt more complex and stable network structure in their gut microbiota than that of the infants with eczema.

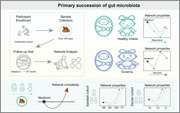

## INTRODUCTION

Eczema (also known as atopic dermatitis, AD) is an immune‐related inflammatory skin disease that frequently develops early in life and lasts into adulthood [1]. According to the Global Burden of Disorder survey, the disease affects at least 15% of children [2], and its prevalence is rising yearly [3]. Symptoms of the disease are related to age, disease stage, ethnicity, and geographic location. In infants, eczema commonly affects the head, face, and extremities, resulting in dry, cracked, and scaly skin.

The immune imprinting can be traced back to the fetal period, and correlated to the maternal microbiota and its metabolites [4, 5], such as short‐chain fatty acids [6], which have been proven to play a significant role in immunity modulation. Microbial composition is closely associated with human health, and the newborns are thought to benefit from exposure to a wide range of microbial organisms [7]. Early microbial colonizers reside in the gastrointestinal system from birth, followed by rapid increases of microbial diversity and community [8]. In the first 100 days of life, infants receive nutrients primarily from maternal breast or formula milk. With respect to that, a large‐scale meta‐analysis has found that children with a family history of atopy are less likely to develop AD as children if they are exclusively breastfed for the first 3 months of life [9]. Given the observation between the microbial diversity and human health, such as immune imprinting and development, understanding the formation and maintenance of microbial communities is necessary for interventions targeting the entire microbial community rather than enrichment or elimination of a single species [8].

Microbiota effects on host physiology were widely studied in four main themes, including barrier function, immune modulation, colonization resistance, and development [10]. Accounting for the beneficial microbial products and functions, the stability of beneficial commensal microbes is thought to be essential for host health and homeostasis [11]. Therefore, drastic changes in the gut microbiota are often associated with disease [10]. Additionally, there are a wide variety of bacterial interactions within the gut ecosystem. By exchanging metabolites, energy, signals, and other materials within the ecological niche, various microbes form a complex ecosystem. To decipher the ecosystem, network algorithms and graph theory have been implied to reveal the competition, co‐occurrence, and interaction within the microbial community [12, 13].

Although several human studies have also reported an association between alterations in the gut microbiota and the prevalence of eczema of children [14, 15, 16], leading to an insightful understanding of microbiome dysbiosis in eczema, there has not been much research investigated on how network structures change in early life. Longitudinal analysis of fecal samples, collected from infancy through early childhood, has enabled the dynamic analysis of early life microbiota. However, the sampling time point selection did not mainly focus on the first 100 days of life, which are crucial for immunological imprinting. Additionally, there is a growing number of evidence that maternal prepregnancy body mass index (BMI) has a limited impact on the development of eczema in offspring [17, 18], while others hold opposing views [19]. Thus, it is still unknown how maternal obesity status is linked to eczema or gut microbiota community. Consequently, it is necessary to conduct additional research to explore the relationship between the gut microbiome and the risk of eczema using advanced and complex methodologies, such as network and graph theory. Further investigation will help us understand the role of the gut microbiota in the onset of eczema in infancy and pave the way for potential translational prospects for the treatment and prevention of this widespread disease.

To better understand the gut microbial network of infants in their first 100 days, we analyzed the 16S rRNA gene amplicon sequencing data of feces from infants recruited at Zhujiang Hospital in Guangzhou, collected at birth, first, second, and third months of age, to explore potential microbiota factors related to eczema. In the present investigation, we explored the pattern and impact of early life microbial network in short‐term and long‐term consequences on children's health. Further studies on these microbial features may facilitate intervention elsewhere and are crucial to understand the underlying mechanisms of the gut microbiota‐disease associations.

## RESULTS

### Basic characteristics

**Figure 1:**
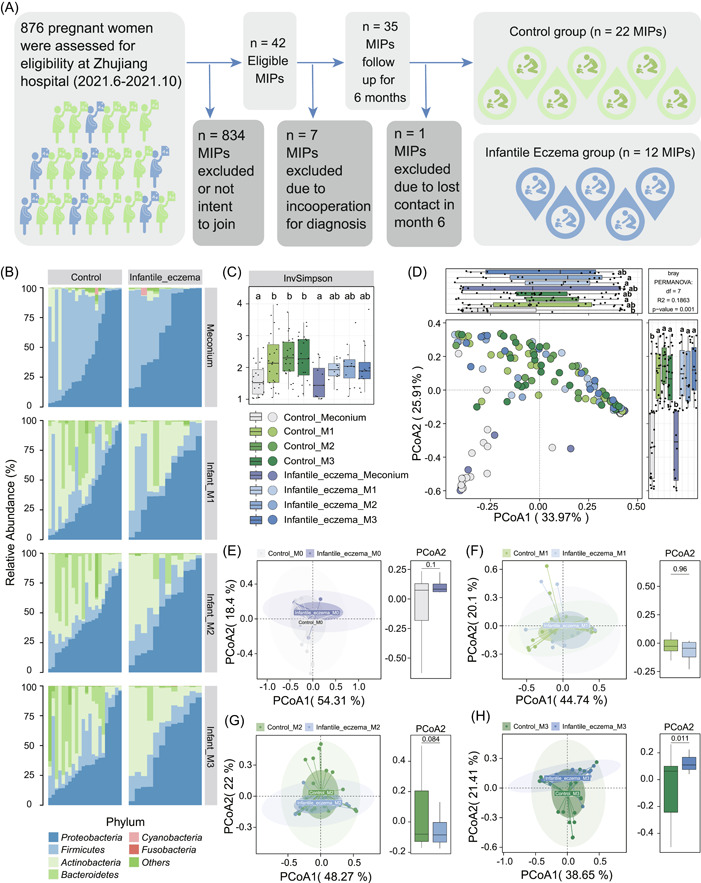
Gut microbial profiling in infancy. (A) The flow scheme of the study population. A total of 34 MIPs were included in the current study after removing all participants who did not meet the predetermined criteria for enrollment and exclusion. (B) Relative abundance of bacterial phyla in the meconium, first‐, second‐, and third‐month fecal samples of infants with and without eczema. (C) InvSimpson index of fecal microbiota. (D) Principal component analysis (PCoA) of Bray–Curtis distance at a family level among samples collected at four time points. Permutation multivariate analysis of variance (PERMANOVA) test was used to assess samples between infants with and those without eczema. (E–H) PCoA plots comparing samples collected at birth (E), first (F), second (G), and third (H) months from infants with and without eczema of β‐diversity were measured by the Bray–Curtis distance at the family level. Significance among multiple groups was tested using a one‐way analysis of variance (ANOVA), followed by the least significant difference (LSD) post hoc test. Groups with different characters denoting a significant difference, while having the same character denotes *p* > 0.05 in the LSD test. Significance between two independent groups was measured by the two‐tailed, unpaired Student's *t* test. InvSimpson, Inverse Simpson; MIP, Mother–Infant Pair.

The flowchart depicted the subject's recruitment and exclusion process (Figure 1A). In the obstetrics and gynecology department, newborns without any abnormalities generally do not undergo any phlebotomy or diagnostic imaging tests, so we only performed routine examinations on the infants, and the results are shown in Table 1. Infants' sex, birth weight, body length, head girth, fetal delivery pattern, and apgar score were not significantly different between the control and case groups. All infants were exclusively or predominantly breastfed and none of the Mother–Infant Pairs (MIPs) received antibiotics in the sampling periods. All 34 mothers enrolled are residents of Guangzhou and had regular maternity checkups during their pregnancies. All mothers received a comprehensive physical examination after admission to the hospital to prepare for delivery, including blood routine examination, coagulation, liver and kidney function, oral glucose tolerance test, infectious diseases, blood type, thyroid function, and glycosylated hemoglobin level. These regularly checked clinical indexes including the prepregnancy BMI and weight gain during pregnancy were not showing a significant difference between control and case groups. However, the maternal predelivery BMI of the case group was significantly lower than that of the control group (Table 1; Supporting Information Table S1). Previous studies have indicated that prepregnancy BMI and pregnancy weight gain have been shown to be associated with the occurrence of perinatal complications and adverse pregnancy outcomes [20, 21]. In the present study, we did not observe a significant difference in maternal prepregnancy BMI but a significant difference in maternal predelivery BMI between two groups. It is worth exploring the underlying link between maternal physical data before delivery and infants' health. Moreover, mothers in the case group had significantly higher blood creatinine and triglyceride (TG) levels before delivery. Creatinine measured in both groups of mothers was within the normal range (Supporting Information Table S1). However, TG was higher than the normal range in both groups of mothers because of hormonal changes during pregnancy which can lead to physiological increases in TGs. High maternal TG levels in late pregnancy may be related to adverse outcomes [22].

**Table 1:** Basic characteristics of the included Mother–Infant Pairs.

	(ALL)	Control *n* = 22	Infantile_eczema *n* = 12	*p* Value
*Infants' characteristics*				
Gender				0.073
Female	17 (50.0%)	14 (63.6%)	3 (25.0%)	
Male	17 (50.0%)	8 (36.4%)	9 (75.0%)	
Birth weight (kg)	3.24 (0.31)	3.21 (0.31)	3.28 (0.30)	0.579
Height (cm)	50.4 (1.41)	50.2 (1.54)	50.6 (1.16)	0.455
Fetal position				0.372
LOA	30 (88.2%)	18 (81.8%)	12 (100%)	
LOT	1 (2.94%)	1 (4.55%)	0 (0.00%)	
ROA	3 (8.82%)	3 (13.6%)	0 (0.00%)	
Head girth (cm)	33.0 [32.0; 33.9]	33.0 [32.0; 33.4]	33.0 [32.0; 34.0]	0.569
Apgar 1 min				1
8	2 (5.88%)	1 (4.55%)	1 (8.33%)	
9	3 (8.82%)	2 (9.09%)	1 (8.33%)	
10	29 (85.3%)	19 (86.4%)	10 (83.3%)	
Apgar 5 min				0.353
9	1 (2.94%)	0 (0.00%)	1 (8.33%)	
10	33 (97.1%)	22 (100%)	11 (91.7%)	
Apgar 10 min: 10	34 (100%)	22 (100%)	12 (100%)	.
Feeding status				0.882
Exclusive breastfeeding	15 (44.1%)	9 (40.9%)	6 (50.0%)	
Predominant breastfeeding	19 (55.9%)	13 (59.1%)	6 (50.0%)	
*Maternal characteristics*				
Age (years)	30.0 [28.0; 33.0]	29.5 [28.0; 33.8]	30.0 [28.8; 31.5]	0.914
Prepregnancy BMI (kg m^−2^)	20.7 (2.35)	21.1 (2.41)	19.8 (2.06)	0.104
Predelivery BMI (kg m^−2^)	26.0 (2.95)	26.8 (2.82)	24.7 (2.77)	0.043
Weight gain (kg)	13.7 (4.97)	14.6 (4.63)	12.0 (5.34)	0.168
Fasting glycaemia (mmol L^−1^)	4.38 (0.29)	4.39 (0.33)	4.34 (0.21)	0.599
OGTT glucose1h (mmol L^−1^)	7.48 (1.61)	7.58 (1.55)	7.28 (1.78)	0.64
OGTT glucose2h (mmol L^−1^)	6.54 (1.12)	6.56 (1.16)	6.49 (1.11)	0.854
TC (mmol L^−1^)	6.16 (1.20)	6.34 (1.33)	5.84 (0.90)	0.226
TG (mmol L^−1^)	2.82 (1.09)	2.52 (0.98)	3.37 (1.11)	0.047

*Note*: Nonnormally distributed continuous variables were presented as Median (IQR); normally distributed continuous variables were presented as Mean (SD). Data are *n* (%) unless otherwise specified.

Abbreviations: BMI, body mass index; IQR, interquartile range; IQR, interquartile range; LOA, left occiput anterior; LOT, left occipitoposterior; OGTT, oral glucose tolerance test; ROA, right occiput anterior; SD, standard deviation; TC, serum total cholesterol; TG, triglyceride.

### Gut microbial diversity differs between healthy infants and those with eczema

The stacked bar plot graphs of the phylum‐level microbiota of the control and case groups are shown in Figure 1B. The dominant early colonizers in the infant gut were Proteobacteria, Firmicutes, Actinobacteria, and Bacteroidetes. Among which, Proteobacteria and Firmicutes, represented in blue, predominated in infant meconium samples, while Proteobacteria and Actinobacteria predominated in postnatal first‐, second‐, and third‐month fecal samples. Alpha diversity was applied to profile the microbial richness and evenness of the gut ecosystem. As shown in Figure 1C, the Inverse Simpson (InvSimpson) indexes of the control group specimens sampled in the first‐, second‐, and third‐month postpartum were significantly elevated compared with that of the fetal meconium. In contrast to the control group, the InvSimpson index of the case group tended to increase compared with the meconium samples in early life, but did not reach a statistical difference (Figure 1C). The microbiota composition was then assessed with the Principal Coordinate Analysis (PCoA), showing that comparing with the microbial structure at the meconium stage, the structure of the gut microbiota at first, second, and third months was significantly different from that of the meconium (Figure 1D). The result echoes the one observed in the stacked bar plot graphs of the bacterial relative abundance (Figure 1B), in which we can also observe apparent dissimilarities between meconium and fecal samples collected at first, second, and third months. To further validate the between‐group variability of microbial structure in samples collected at different time points, we performed PCoA. As shown in the results, there were no significant differences in the gut microbial structure between the two groups of infant meconium samples (Figure 1E, *p* = 0.1) and fecal samples at first‐month postpartum (Figure 1F, *p* = 0.96) and at second‐month postpartum (Figure 1G, *p* = 0.084). From the intergroup variability analysis in the PCoA axis 2, we have demonstrated that the gut microbiota structure had a tendency to separate between groups along with the growth of infants and maturation of gut microenvironment. It was interesting to observe significant differences in axis 2 of the PCoA at the third‐month postpartum (Figure 1H, *p* = 0.011). Together, these results suggest that the composition of the gut microbiota gradually changes during infancy, and the extent of increase in gut microbiota richness is more significant in control group infants.

### Healthy infants share similar rhythmicity in early life microbial network

**Figure 2:**
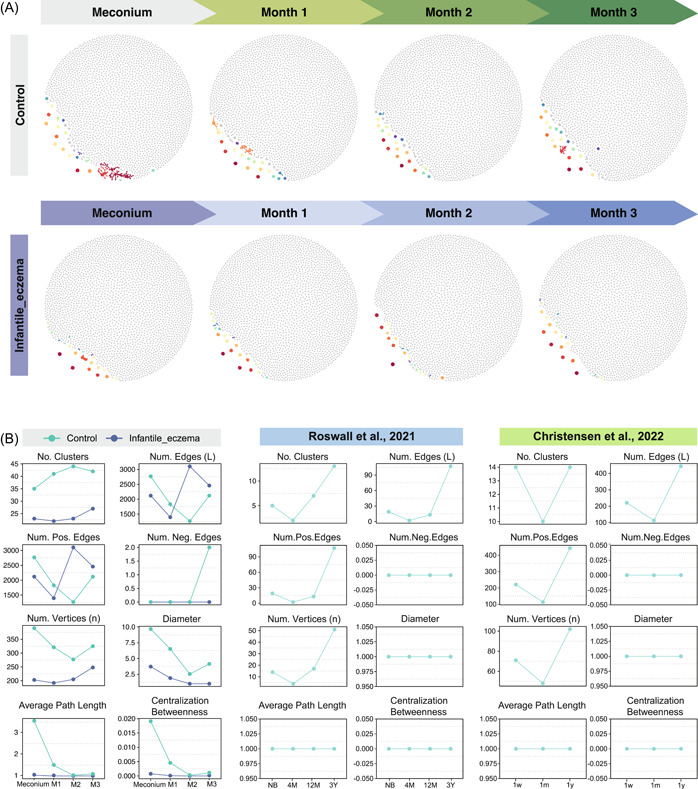
Dynamics of fecal microbial networks in infancy. (A) Visualization of the constructed networks, depicting early colonizers in the first 100 days. The networks in the first row were constructed based on the gut microbiota of the control group; the networks in the second row belong to the case group (Infantile eczema group). Each row contains four networks, showing the structure of gut microbiota at birth, first‐, second‐, and third‐month postpartum. Modules with ≥2 nodes are presented in different colors, and the rest nodes are presented in gray. (B) Dynamic changes in network topology, including Number of clusters (No. Clusters), Number of edges (Num. Edges), Number of positive edges (Num. Pos. Edges), Number of negative edges (Num. Neg. Edges), Number of Vertices, Diameter, Average path length, and Centralization betweenness. Green symbols represent the network properties of infants without eczema, including infants in the control group and healthy infants from the studies of Roswall et al. and Christensen et al. The blue symbols represent the network properties of the case group.

With the increasing importance of the complexity of the ecological network affecting the ecosystem stability, our understanding of gut microbiota network of newborns is still in its infancy. To better demonstrate the structure of the gut microbiota network in early life, we obtained stool samples from infants, using a sampling interval of 1 month. We constructed time‐series molecular ecological networks (MENs) based on the amplicon sequencing variants (ASVs) at the family level of each set of samples (Figure 2A). These findings were further corroborated by randomly selecting 16 healthy infants from the Swedish cohort in the study of Roswall et al. [23] and the Danish cohort in the study of Christensen et al. [24]. Longitudinal samples collected at different time points were obtained, and MENs were constructed (Supporting Information Figure S1A,B), from which we can also observe interactive networks existing in gut microbiota in early life. Network topological indexes were used to demonstrate visual mapping, including Number of clusters (No. Clusters), Number of edges (Num. Edges), Number of positive edges (Num. Pos. Edges), Number of negative edges (Num. Neg. Edges), Number of Vertices, Diameter, Average path length, and Centralization betweenness, among which several indexes were greater than zero in the fetal meconium sample, indicating a complex network of microbiota existing in gestation period (Figure 2B).

On the basis of the results, we can observe an increased complexity in the network topology of early colonizers in healthy infants, such as No. Clusters, Num. Edges, Num. Pos. Edges, Number of Vertices, Average degree (Average K), Relative modularity (RM), and Centralization degree (Figure 2B; Supporting Information Figure S1C). From the line charts in green color, we can observe a gradual increase in these indexes from the second sampling time points, suggesting a trend of complexity and maturity of the networks over time. In the independent cohorts from the studies of Roswall et al. [23] and Christensen et al. [24], certain network topological indexes remain zero, such as Diameter, Average path length, and Centralization betweenness, but a trend of changes can be observed in the infants without eczema in the current cohort. We hypothesize that this is due to the sequencing quality differences in 16S rRNA data. In the current study, we have obtained high‐quality sequencing data due to the application of sophisticated sampling and sequencing protocol as well as sequencing machine. On the contrary, 16S rRNA data from independent cohorts were subjected to quality control and the unqualified sequences were filtered in the process of upstream data analysis, resulting in a limited number of ASVs for downstream analysis. To further explore the fundamental microbial characteristics of independent cohorts, we investigated the top 10 most abundant taxa, alpha diversity as well as beta diversity of independent data sets (Supporting Information Figure S2). From these figures, we were able to draw the conclusion that while the gut microbial characteristics of healthy infants vary across the three data sets, the network topological indexes were at a high level in samples taken from newborns, and an increased trend of network complexity can be seen along with the maturity of the gut microbiota. Notably, the number of keystone nodes index of all three batches of time‐series data resulted in the number zero, reflecting that although a sparse network structure had formed in the gut early in life, there were still no keystone core bacteria that could meet the requirements for the assessment of this index. Furthermore, from the data we have obtained, Num. Neg. Edges can be observed in the third month, reflecting a competition among the early colonizers. Together, these data illustrate that the network topological indexes were higher in samples taken from newborns, and an increased trend of network complexity can be observed along with the maturity of the gut microbiota. The above findings demonstrate that healthy infants have rhythmic changes in the gut microbial network, which may be essential for healthy developments in early life.

### Healthy infants have a denser gut microbiota network compared with that of infants with eczema

The microbiota has internal interactions and plays a pivotal role in the onset and etiology of eczema [11, 25, 30]. It is therefore vital to identify interactive networks within groups and establish the pattern of microbiota interaction within the 100 days after birth. Previous studies have explored multiple aspects of gut microbiota composition in infants presenting with eczema. In the present study, the number of clusters formed in the meconium samples of the control group was higher than that of the infants in the case group during the same period (Figure 2B). Additionally, the number of clusters in the control group increased at the beginning, followed by a slight decrease in the third month, but still remained higher than that of the first month and meconium. For the case group, the number of clusters decreased primarily after birth and then significantly increased in the third month. The number of edges and vertices in the network diagram of this study decreased at the beginning and then increased in the control group, and these dynamic patterns were also verified in the independent data sets. In the control group, the index increased in the third month, while it leveled off in the case group. The diameter index did not change in this mapping mode due to the small number of vertices obtained in the independent data sets. Another interesting observation is that negative edges, reflecting the emergence of competing antagonistic clusters in microbiota communities, could be observed in the control group by the third month of the study, but not in the case group. Nevertheless, the blue curves in the line charts, representing the case group, show an arrhythmic pattern in the first 100 days of life. For example, Num. Edges, Num. Pos. Edges, Connectance (Edge density), Average degree (Average K), Centralization closeness, and Centralization degree presented a peak in the second month, followed by a rapid decrease in the third month, which may be correlated with the onset of infantile eczema (Figure 2B; Supporting Information Figure S1C). In summary, we have identified a loss of rhythm in network signatures in infants with eczema, whereas healthy infants have a denser gut microbiota network.

### Establishment of early microbial colonizers in infants with and without eczema

**Figure 3:**
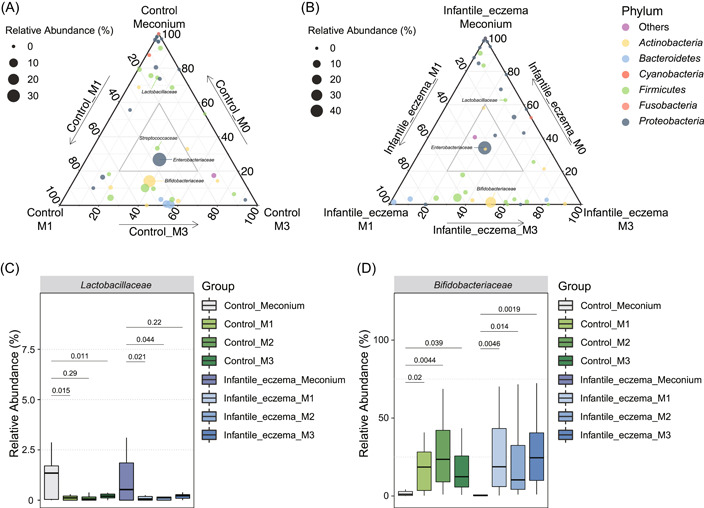
Establishment of early colonizers. (A) Ternary plots of early colonizers at birth, first, and third months of control group. (B) Ternary plots of early colonizers at birth, first, and third months of Infantile eczema group. Dots were taxa at the family level and colored according to their phylum level. (C) Relative abundance of Lactobacillaceae. (D) Relative abundance of Bifidobacteriaceae. Significance between two independent groups was measured by the two‐tailed, unpaired Student's *t* test.

Strain‐level study found that vertical transmission of strains between mother and infant is the main source of the first colonizers in infant gut [26]. Given the drawbacks of 16S rRNA gene amplicon sequencing in identification accuracy at species level, in the current study, we compared the differences between samples at different time point groups at the family level, but did not observe any differential bacteria, which is likely due to the fact that the infants recruited for this study were healthy vaginally delivered and had very similar diets in the first 3 months of life, breastfed exclusively or predominantly. Notably, predominant breastfed infants receive occasional supplements for the formula. To probe the development of early colonizers in the infant gut, ternary diagrams were applied to represent the biomass conversion processes (Figure 3A,B). *Enterobacteriaceae* and *Bifidobacteriaceae* are among the top dominant bacteria at the family level in both control and case group samples at birth, first‐ and third‐month postdelivery (Supporting Information Tables S2 and S3). From the ternary plots, we can also observe drastic changes of gut microbiota in the first 100 days, with only 2 and 5 dominant families lasting from birth to the third month in the control and case groups, respectively (Figure 3A,B). *Lactobacillaceae* is richer at birth (Figure 3A,B), though its relative abundance is lower than 1% other than meconium (Figure 3C,D). *Enterobacteriaceae* are a family of facultative anaerobic bacteria, which are one of the commensals in humans and animals. On the other hand, two prominent probiotic families, *Bifidobacteriaceae* and *Lactobacillaceae*, contain well‐known probiotics, such as *Bifidobacterium longum*, *Bifidobacterium animalis*, and *Lactobacillus rhamnosus*, show opposite trends in abundance in early life. These probiotics play a critical role in the development of the infant's immune system [27, 28, 29]. In the present study, we observed that the relative abundance of *Lactobacillaceae* in the infant's feces decreased significantly after the initiation of breastfeeding (Figure 3C). In contrast, the *Bifidobacteriaceae* were more abundant in the gut by the onset of diet (Figure 3D). From the ternary diagrams, we can also conclude that the development patterns of gut microbiota are quite different between infants with and without eczema.

### High maternal TG levels in late pregnancy could be a risk factor for eczema

Using independent *t*, Mann–Whitney, *χ* ^2^, and Fisher's exact tests, if necessary, univariate comparisons between two groups on infants' basic characteristics, maternal demographics, and clinical results were carried out. To identify the variables independently linked with the occurrence of eczema, variables with *p* < 0.05 in the univariate analyses were chosen as potential independent variables for a multivariable logistic regression. Though the statistical result of maternal prepregnancy BMI did not meet the statistical threshold of 0.05, it has a direct impact on predelivery BMI. Thus, prepregnancy BMI was included in multivariable logistic regression analysis. With the inclusion of all outcome variables at once, the statistical results revealed a positive association between the development of eczema and the maternal TG level in late pregnancy (odds ratio adjusted [95% CI] = 3.41 [1.13–10.25], *p* = 0.029) (Table 2). High maternal TG level in late pregnancy could be a risk factor for eczema.

**Table 2:** Multivariable logistic regression.

	ORc (95% CI)	ORadj (95% CI)	*p* Value (Wald's test)	*p* Value (LR‐test)
Prepregnancy BMI	0.76 (0.53, 1.11)	0.87 (0.46, 1.64)	0.662	0.659
Predelivery BMI	0.67 (0.45, 1)	0.62 (0.34, 1.13)	0.12	0.086
TG	2.24 (1, 5.03)	3.41 (1.13, 10.25)	0.029	0.009

Abbreviations: BMI, body mass index; CI, confidence intervals; LR‐test, Likelihood Ratio *χ* ^2^ test; ORadj, adjusted odds ratio; ORc, crude odds ratio; TG, triglyceride.

## DISCUSSION

We collected well‐matched fecal samples at four time points (meconium, first, second, and third months of postpartum [infants]) and completed a regular follow‐up to obtain detailed information of each MIP. On the basis of 16S rRNA gene amplicon sequencing data from three independent longitudinal investigations, we identified that the network topological indexes are at a high level in samples collected from newborns, and an increased trend of network complexity can be seen along with the maturity of the gut microbiota. Furthermore, we showed that healthy infants have a denser gut microbiota network than that of the infants with eczema. In particular, our findings suggest that the composition of the gut microbiota gradually changes during infancy, with healthy newborns showing greater increases in gut microbial richness, and high maternal TG in late pregnancy may be a risk factor for eczema.

There is mounting evidence that the gut microbiota is crucial to the development of the immune system and the prevention of eczema in the periods of neonatal, infant, child, and adult [14, 15, 31, 32, 33–33, 35]. Of these, several cross‐sessional studies [15, 31, 32, 35] have obtained fecal samples of infants below 6 months of age, while others [14, 33] performed longitudinal sampling. A longitudinal study of fecal microbiota of children from 5 weeks through 6–11 years has indicated that alterations in microbial diversity and composition precede the onset of allergic manifestations [14]. Additionally, early colonizers began colonizing the gastrointestinal system at birth, followed by rapid increases of microbial diversity and community [8]. These findings emphasized the importance of microbial succession and maturation in early life, yet the research described above rarely focuses on it. In particular, there are not many fecal samples from the first 100 days of life. Only one study [14] included samples from children aged 6–11 years old and at 5, 13, 21, and 31 weeks postpartum (infants); however, the sampling gap was quite wide and no meconium samples were taken. In our study, with a sampling gap of 1‐month, fecal samples were collected from infants at four time points, allowing us to explore the microbial signatures and maturation during the very early life period. In addition, one recently published research showed no evidence linking the meconium gut microbiota composition to atopic symptoms in children up to age 4 years [34]. In the current study, we did not observe structural difference in meconium samples between groups, but difference can be observed from fecal samples from the second month (Figure 1F, *p* = 0.084) and reach statistical significance in the third month (Figure 1G, *p* = 0.011). In summary, after controlling the confounding factors, including birth mode, breastfeeding status, and antibiotic usage, we have identified aberrant alpha and beta diversities in infants with eczema in the first 100 days of life.

A more comprehensive picture of the status and significance of the microbial network to health can be obtained by exploring the early life microbiota and combining microbial signatures with infants' medical records. In a long history, graph theory has been used to interpret mathematical and theoretical developments. As network research has grown out of social network analysis and network science, graph theory has also started to be applied to these fields. However, studies that seek to investigate the characteristics of the gut microbial network mostly lack further descriptions of network properties. The co‐occurrence network, one of the most used network types, was available and simply displayed the positive and negative association between different taxa [35]. Though co‐occurrence network can display the complex relationship between different taxa, we cannot ignore the fact that a single universal healthy microbiome configuration does not exist [36]. ASVs are pre‐annotated nucleic acid sequences of the microbes, which may be better applied in constructing network structures to demonstrate the interconnectedness within the ecosystem. Further, in a population‐based metagenomics analysis, the authors demonstrated that 16S rRNA gene sequencing surpasses metagenomic shotgun sequencing in predicting microbial families [37]. Combined with network topology indexes, together they demonstrate the complexity and stability of the network. To test our hypothesis that rhythmic dynamic changes in early life are closely related to healthy infancy, we constructed MENs based on the ASVs at the family‐level of three independent data sets, and demonstrated a shared rhythm in network topological indexes among healthy infants and illustrated infants without eczema have a denser gut microbiota network compared with that of the infants with eczema.

The causes of eczema are multifaceted, including genetic, parental, and environmental variables. Among these factors, the infant's gut microbiome is significant for its impact on the development of the child's immune system. In this study, the InvSimpson index was significantly elevated in healthy infants in early life, while infants with eczema could only observe a trend of elevation but not statistically significant (Figure 1B). The result indicates the InvSimpson index may be a protective factor, which is in line with a previous study [15]. InvSimpson index is one of the alpha‐diversity indexes that can present the richness and evenness of the microbiome. One possible reason may be the difference in living environment between the two groups of infants. An earlier study found that infants with a higher‐level education father had a higher risk of having eczema (odds ratio adjusted [95% CI] = 9.93 [1.83–53.71], *p* = 0.008) [32]. The researchers also speculated that fathers with higher levels of education may have a better understanding of hygiene, which suggests a lower incidence of infections transmitted through contact with unsanitary environments in early childhood could be a cause of the rise in allergic diseases. Another study investigated the impact of urbanized microbiota in infants, immune constitution, and later risk of atopic diseases, illustrating changes in the infant microbiota caused by urbanization may increase the risk of asthma and atopic traits, most likely through interactions with the developing immune system [16]. A growing number of studies have investigated the beneficial effects of farming lifestyle [38], claiming that due to environmental changes, the gradual loss of certain microbial exposures contributes to the global rise of allergic diseases. Though the infants in the current study were born and raised in an urbanized city, Guangzhou, China, their living environment may still be different. The aforementioned findings imply that boosting gut microbial diversity by exposure to nonurbanized areas may be a method to reduce the risk of acquiring allergy disorders. In the current study, from the indexes of alpha diversity and network properties, we can observe that the development of gut microbiota in infants with eczema was aberrant. This may suggest that the richness of early colonizers in the gut may be important for developing complex microbial networks. Given our observation that emerging scientific research has demonstrated health benefits of prebiotics, probiotics, and synbiotics on the host [39], we speculate that supplementation of probiotics, prebiotics, and synbiotics could be sufficient to drive microbial network progression via increasing the richness of gut microbiota. Other than the InvSimpson index, which may be closely related to environmental factors, the current study has found that high maternal TG in late pregnancy may be a risk factor for eczema (odds ratio adjusted [95% CI] = 3.41 [1.13–10.25], *p* = 0.029). Though maternal predelivery BMI was significantly different between the two groups, the analysis of multivariable logistic regression has elucidated that only high maternal TG in late pregnancy was the risk factor related to the onset of eczema. In the previous studies, some found maternal obesity and gestational weight gain are not risk factors of atopic disease [17, 18], while there is also a report of maternal prepregnancy BMI being one of the risk factors for eczema in childhood [19]. Further investigation is needed to further elucidate the underlining mechanism.

This study has certain limitations. First of all, it is not a population‐based study, further efforts should be made in the same research field. Seasonal variations and different climate factors may have an impact on the prevalence and severity of eczema [40]. Though infants in the current study were recruited in summer in Guangzhou, China, when temperatures were around 30°C, we did not do a survey on the infants' living environment. Furthermore, eczema was suggested to be associated with impaired sleep quality throughout childhood [41], indicating the importance of long‐term tracking on the participants' disease stage, simultaneous symptoms, as well as sleep quality, and sleep duration. Last but not least, a daily sustained sampling interval will provide more insight on network complexity development throughout the process of gut microbial primary succession.

## CONCLUSION

Network analysis and graph theory can be applied to discover the process of microbial formation and construction. In our current investigation, our data demonstrate that healthy infants from different ethnicities share similar gut microbial network changes in early life, while infants with eczema present a less complex and stable network compared with healthy infants. Using longitudinal data, we demonstrated that the microbial structure of infants with eczema was aberrant in the third month. The findings also emphasized the potential protective factor, InvSimpson index of gut microbiota, and the risk factor, high maternal TG levels in late pregnancy, may be correlated with the onset of infantile eczema.

### Materials and methods

#### Participant enrollment

From June 2021 to October 2021, all the pregnant women admitted to the Obstetric Department of Zhujiang Hospital of Southern Medical University in Guangzhou, China, were assessed for eligibility. Previous studies have shown that the mode of birth significantly affects the structure of the neonatal gut microbiota. Infants are exposed to Lactobacillus in the mother's vagina via the vertical transmission route during the vaginal delivery [26]. Hence, we only recruited infants delivered vaginally in the current study. The World Health Organization (WHO) and United Nations International Children's Emergency Fund (UNICEF) recommend that children initiate breastfeeding within the first hour of birth and be exclusively breastfed for the first 6 months of life [42]. As a result, pregnant women with the will for exclusive breastfeeding were recruited. Furthermore, with respect to the high interindividual variation, we held to strict criteria during the enrollment (as shown in *Method*), resulting in cohorts of a small size within 4 months period, despite being in one of the top hospitals. In the current study, 876 healthy pregnant women and their babies were screened, of which most of them were excluded because they did not meet the enrollment criteria or refused to participate in this study. The remaining 42 pairs were enrolled. The early colonizers in life are essential for health, especially for the development of the infant's immune system [8]. The (neonatal) meconium is formed mainly during the fetal period, while the fetus is exposed to a relatively anaerobic environment, and the proportion of facultative anaerobes and strictly anaerobic bacteria in meconium samples is significantly higher than that of the infant feces collected later [43]. Since the development of the immune system can be traced back to the embryonic period [8], we hypothesize that fetal stool samples are also associated with the immune formation during the fetal period and the network structure of meconium may be related to the onset of immune‐related disease.

Thus, meconium samples from the infants at the time of delivery were collected in hospital by a doctor. All the mothers received a sample collection handbook on the proper way to collect infant fecal samples of first‐, second‐, and third‐month postpartum at home. We provide health guidance and record basic information, such as health status and medication usage, for the first 3 months postpartum. Infants' health status information was again collected via questionnaire at 6 months of age. However, during the sampling period, seven MIPs withdrew from the study due to refusal to continue or diagnosis of disease. So eventually, we obtained samples from 35 MIPs at four time points and these samples were proceeded with 16S rRNA gene amplicon sequencing. Because one mother did not complete the questionnaire at 6 months postpartum, we grouped the remaining 34 infants according to whether they developed eczema by month 6, resulting in 22 healthy infants (control group) and 12 infants with eczema (case group).

#### Enrollment criteria

Birth mode, maternal antibiotic use, and breastfeeding rank the top influential factor in microbiota development, and we accordingly designed strict inclusion and exclusion criteria to minimize the bias [8, 14, 44]. The inclusion criteria of the current study include: (1) healthy pregnant women aged between 20 and 40; (2) vaginally delivered infants; (3) maternal prepregnancy BMI < 28; (4) maternal will of exclusive breastfeeding; (5) infant's birth weight between 2500 and 4000 g. The exclusion criteria include: (1) gestational diabetes, depression, hypertension, or other diseases; (2) taken probiotics or antibiotics within 3 months before delivery; (3) use of antibiotics during labor; (4) history of gestational smoking and alcohol consumption; (5) diarrhea in the 3 months before delivery; (6) use of antibiotics in the first 100 days after delivery; (7) declined exclusive or predominant breastfeeding in the first 100 days of life; (8) diagnosed of genetic diseases.

Finally, 35 MIPs completed longitudinal sampling and medical consultation. One MIP lost contact in month 6 without filling in the questionnaire. Before the experiment, all participants have been informed of the risks and benefits of the experiment and we have received informed consent with written permission from each mother.

#### Human study populations and metadata

The current cohort consisted of 34 full‐term infants born vaginally. No statistical method was used to predetermine the sample size. However, on the basis of the previous studies [13, 27, 32], we hypothesized that 34 infants with four time points' samples would be adequate to illustrate the dynamics of microbial network. All the recruited infants were born at Zhujiang hospital, with no symptoms of any disease and were breastfed within the first 24 h. Doctors were trained to collect first‐pass meconium sample into sterile tubes, and parents were taught to collect fecal samples from nappies into sterile tubes at first‐, second‐, and third‐month postpartum. Once the sample is collected, tubes together with an oxygen‐absorbing agent paper sachet (MGC AnaeroPack‐Anaero) were kept in a culture bag (MGC AnaeroPack‐Anaero). The culture bag and ice bags were immediately stored inside an insulated box, delivered to State Key Laboratory of Applied Microbiology Southern China for probiotics isolation (e.g., Lactobacillus and Bifidobacterium) and the rest samples were frozen with liquid nitrogen and then stored at −80°C until sample preparation. Health status, pattern of feeding, infant/maternal medication, and general information were collected via medical consultation at first‐, second‐, and third‐month postpartum, and questionnaire at 6 months postpartum. Though infants are mostly breastfed, some occasionally received formula for certain reasons, including shortness of breast milk, mother was not around the infant at dining time, and nutritional supplementary. On the basis of the history of the medical consultation and the results of questionnaire, we classified samples of infants who did not ever develop eczema by 6 months into the control group, and the rest into the infantile eczema group. Of all the participants, only one infant from the control group had a family history of eczema.

The validation cohort of Roswall et al. [23] consisted of a random subset of 16 healthy Swedish infants from the original cohort. Well‐matched fecal samples collected at birth, fourth months, and first and third years were generated for network analysis. The validation cohort of Christensen et al. [24] also consisted of a random subset of 16 healthy Danish infants from the original cohort. Well‐matched fecal samples collected at birth, 1 month, and 1 year were generated for network analysis.

#### Gut microbiota analysis

Following the manufacturer's instructions, DNA was extracted from samples using the E.Z.N.A. Stool DNA Kit (D4015, Omega Inc.). Using the primers 341F (5′‐CCTACGGGNGGCWGCAG‐3′) and 805R (5′‐GACTACHVGGGTATCTAATCC‐3′) [45], the bacterial and archaeal 16S rRNA gene's V3–V4 region was amplified.

A total of 16S rRNA amplicons were then sequenced using an Illumina NovaSeq platform in accordance with LC‐Bio's instructions for use. Paired‐end reads were merged using FLASH. Denoise with dada2 (generate ASV tables), feature data summaries, taxonomic analysis (mapping reads to Greengenes 13_8), and generation of taxonomic tables were done according to the QIIME2 (Version 2022‐2) [46]. Taxonomic tables were decontaminated by eliminating the low abundant taxa (mean relative abundance < 0.1%; prevalence < 10% within each group). Filtered taxonomic tables were used for microbial analysis. Network analysis was carried out with an R package ggClusterNet (Version 0.1.0) [47], with model_igraph (method = “cluster_fast_greedy”) function. Alpha diversity, beta diversity, PCoA, permutation multivariate analysis of variance test, and ternary analysis were performed with home‐developed microbial analysis R package EasyMicroPlot (Version 0.5.1) [48].

### Sampling and sequencing characteristics

Considering that the meconium is biological samples with low microbial concentrations and is more susceptible to external interferences, we referred to the sample collection method used in previous studies with minor customization on the sampling procedure (as shown in Participant enrollment). Finally, 140 collected infant fecal samples were sequenced on the Illumina NovaSeq platform, among which 136 samples were used to interpret connections between microbial networks and eczema. After joining the paired‐end sequencing files, we can see that the mean of sequenced reads of each sample is 88,405, with the minimum value of 54,615 and the maximum value of 124,965.

### Statistical analyses

All data analyses were conducted in R version 4.2.1. To explore the network signatures, we perform Spearman correlation and Benjamini–Hochberg method to adjust *p* value with an R package ggClusterNet (Version 0.1.0). In network analysis, all molecular networks were constructed on the basis of Spearman correlations of relative ASV abundances, the threshold for Benjamini–Hochberg adjusted *p* value was 0.05, and the absolute value of correlation coefficients should be over 0.3. Before the statistical analysis, a Shapiro–Wilk test was conducted to verify the normality of measurements. Depending on the distribution of the variables, either the Mann–Whitney test or the unpaired Student's *t* test was employed for quantitative variables. The Kruskal–Wallis test for mean values was used to evaluate nonparametric comparisons between participants in all groups. Significance among multiple groups was tested using a one‐way analysis of variance, followed by the least significant difference (LSD) post hoc test. Groups with different characters denotes a significant difference, while having the same character denotes *p* > 0.05 in the LSD test.

## AUTHOR CONTRIBUTIONS

Liwei Xie and Ying Ma designed the study and acquired grants. Kai Ma, Runxin Wang, and Guangxian Wang on behalf of the members from Jiangsu New‐bio Biotechnology Co., Ltd., which provided the industrial seeding grant to support the basic and clinical studies presented in current investigation. Liujing Huang, Guihua Pan, Yifei Fenga, Zijing Fan, Guangye Huang, Sixia Huang, Yuhui Hou, and Mulan Han collected and analyzed the data. Liujing Huang and Liwei Xie drafted and edited the manuscript, and accessed and verified the underlying data reported in the manuscript.

## CONFLICT OF INTEREST STATEMENT

The authors declare no conflict of interest.

## ETHICS STATEMENT

All research‐related protocols were approved by the Medical Ethics Committee of Zhujiang Hospital of Southern Medical University and registered at clinicalTrials.gov (NCT05462366).

## Supporting information

Supporting information.

Supporting information.

## Data Availability

All 16S rRNA gene amplicon sequencing data were deposited in the National Microbiology Data Center (NMDC, https://nmdc.cn/) under the BioProjects NMDC10018172. The validation 16S rRNA gene amplicon sequencing data from the studies of Roswall et al. and Christensen et al. are listed in Supporting Information Tables S4 and S5, respectively. The code scripts used for data processing, analysis, and visualization have been deposited at GitHub (https://github.com/xielab2017/EasyMicroPlot) [48]. Upon request, additional information is available from the corresponding author
