# Supplementary material for: Microbial network signatures of early colonizers in infants with eczema
Source: Imeta. 2023 Feb 16;2(2):e90. doi: 10.1002/imt2.90 (PMC10989766; doi:10.1002/imt2.90)
Supplement: Supplementary file 1 — Supporting information. [file IMT2-2-e90-s002.docx]

## Microbial network signatures of early colonizers in infants with eczema

**Running title**: Infant microbial network signatures of eczema

Liujing Huang^a,b,#^, Guihua Pan^b,#^, Yifei Feng^a^, Zijing Fan^b,c^, Kai Ma^d^, Runxin Wang^d^, Guangxian Wang^d^, Guangye Huang^a^, Sixia Huang^a^, Yuhui Hou^a^, Mulan Han^b^, Liwei Xie^a,b,c,*^, Ying Ma^a,*^

^a^Obstetrics and Gynecology Medical Center, Zhujiang Hospital, Southern Medical University, Guangzhou 510280, China

^b^Guangdong Provincial Key Laboratory of Microbial Culture Collection and Application, State Key Laboratory of Applied Microbiology Southern China, Institute of Microbiology, Guangdong Academy of Sciences, Guangzhou 510070, China

^c^School of Public Health, Xinxiang Medical University, Xinxiang 453003, China

^d^Jiangsu New-bio biotechnology Co., Ltd, Jiangyin 214437, China

^*^Correspond authors：[xielw@gdim.cn](mailto:xielw@gdim.cn) (Liwei Xie), [mayingwuzhuoyi@126.com](mailto:mayingwuzhuoyi@126.com) (Ying Ma)

^#^Liujing Huang, and Guihua Pan contributed equally to this article. The author order was determined by their equal but gradated contributions for this paper.

Supplementary Figures


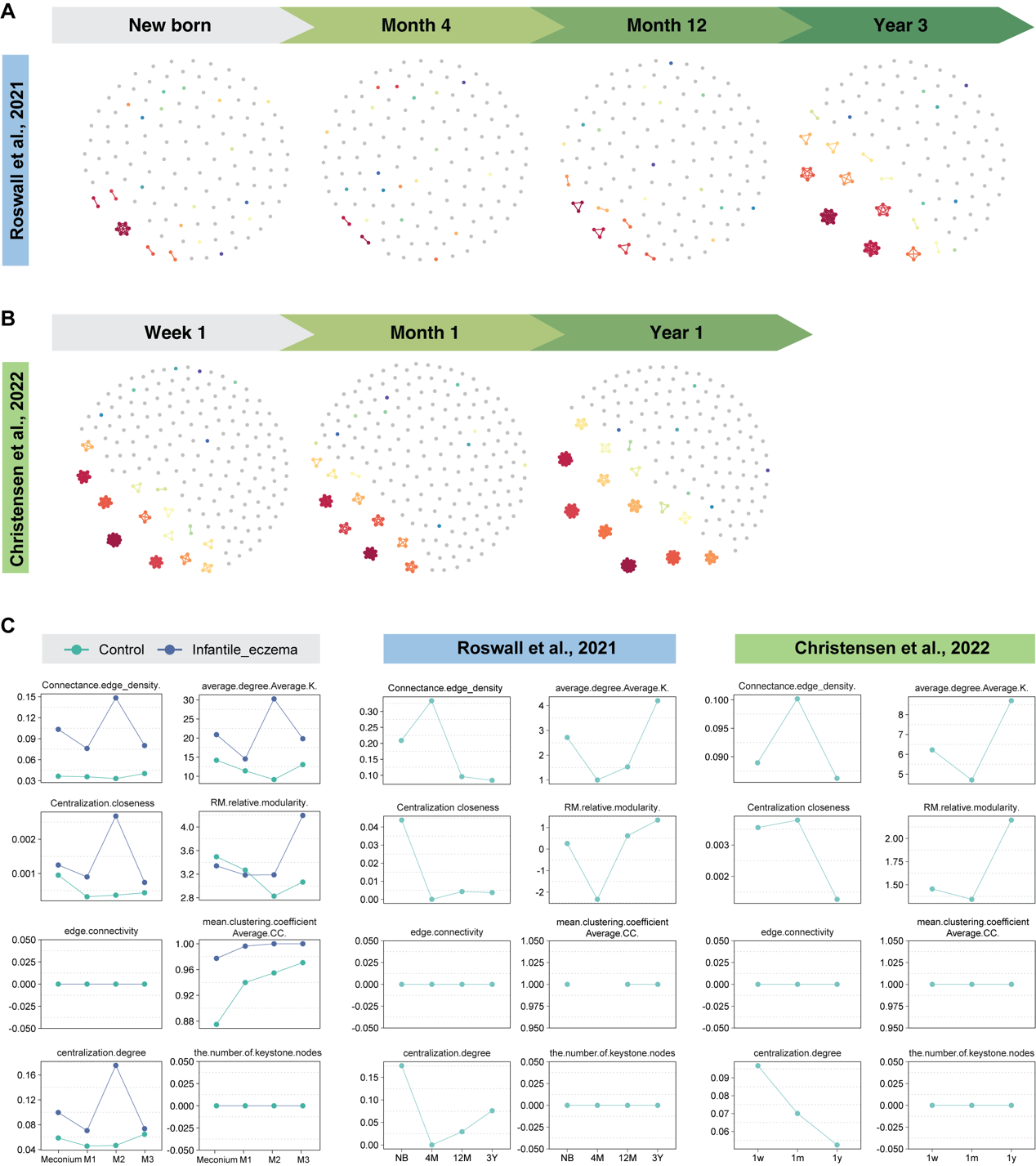


**Supplementary Figure 1. Dynamics of fecal microbial networks in infancy**. (A-B) Visualization of the constructed networks, depicting early colonizers in the infancy through childhood. The networks (A) in the first row were constructed based on the gut microbiota of the healthy infants from the study of Roswall et al.; the networks (B) in the second row belong to the healthy infants from the study of Christensen et al. Modules with ≥2 nodes are presented in different colors, and the rest nodes are presented in grey. (C) Dynamic changes of network topology, including Connectance (Edge density), Average degree (Average K), Centralization closeness, Relative modularity (RM), Edge connectivity, Mean clustering coefficient (Average CC), Centralization degree, and the Number of keystone nodes. Green symbols represent network properties of the infants without eczema, including infants in the Control group and healthy infants from the studies of Roswall et al. and Christensen et al. The blue symbols represent network properties of the Case group


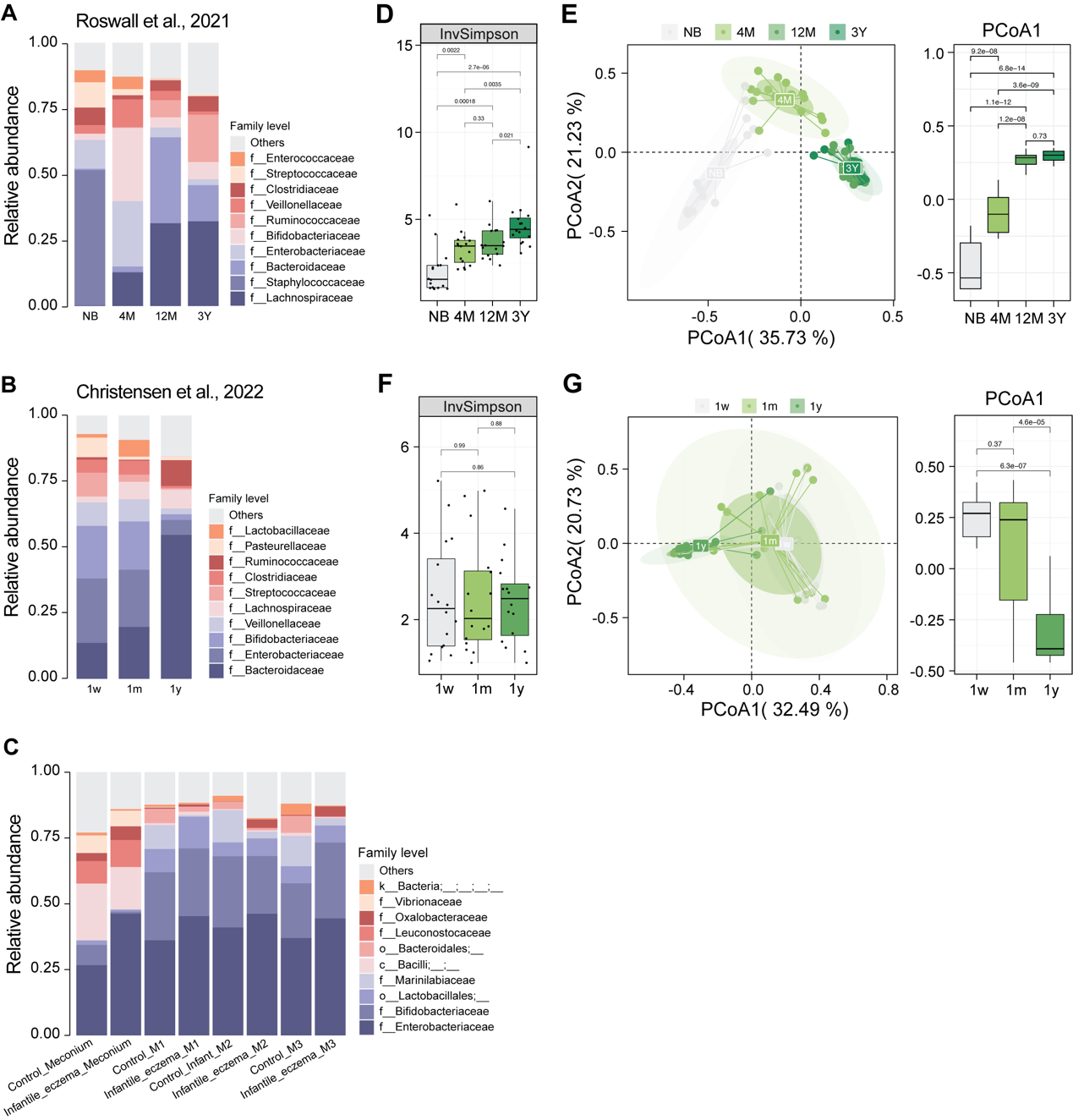


**Supplementary Figure 2. Gut microbial profiling of the independent datasets.** (A-C) Relative abundance of bacterial family in the fecal samples of infants from different cohorts. (D) InvSimpson index of fecal microbiota in samples derived from the study of Roswall et al. (E) Principal component analysis (PCoA) of Bray-Curtis distance at family level among samples collected at 4 time points. (F) InvSimpson index of fecal microbiota in samples derived from the study of Christensen et al. (G) Principal component analysis (PCoA) of Bray-Curtis distance at family level among samples collected at 3 time points. Significance among multiple groups was tested using a one-way analysis of variance (ANOVA), followed by the least significant difference (LSD) post hoc test. Groups with different characters denoting a significant difference, while having the same character denotes *p*-value > 0.05 in LSD test. Significance between two independent groups was measured by two-tailed, unpaired Student's t test.

**Supplementary Table in excel**

Supplementary Table 1 Basic characteristics of the mothers

Supplementary Table 2 Ternary results of control group

Supplementary Table 3 Ternary results of case group

Supplementary Table 4 Metagenomes and metadata used to validate the dynamic changes in early life. The validation 16S rRNA gene amplicon sequencing data from the study of Roswall et al.

Supplementary Table 5 Metagenomes and metadata used to validate the dynamic changes in early life. The validation 16S rRNA gene amplicon sequencing data from the study of Christensen et al.
